# Recent Advances in the Treatment of Coronary In-Stent Restenosis

**DOI:** 10.31083/j.rcm2512433

**Published:** 2024-12-06

**Authors:** Luca Sartore, Mauro Gitto, Angelo Oliva, Ryota Kakizaki, Roxana Mehran, Lorenz Räber, Alessandro Spirito

**Affiliations:** ^1^Department of Cardiology, Bern University Hospital, Inselspital, CH-3010 Bern, Switzerland; ^2^Mount Sinai Fuster Heart Hospital, Icahn School of Medicine at Mount Sinai, New York, NY 10029, USA

**Keywords:** in-stent restenosis, drug eluting stent, drug coated balloon, atherectomy, cutting balloon, scoring balloon, intravascular lithotripsy, intravascular brachytherapy, optical coherence tomography, intravascular ultrasound

## Abstract

In-stent restenosis (ISR) remains the predominant cause of stent failure and the most common indication for repeat revascularization. Despite technological advances in stent design, ISR continues to pose significant challenges, contributing to increased morbidity and mortality among patients undergoing percutaneous coronary interventions. In the last decade, intravascular imaging has emerged as an important method for identifying the mechanisms behind ISR and guiding its treatment. Treatment options for ISR have expanded to include balloon angioplasty, cutting or scoring balloons, intravascular lithotripsy, atheroablative devices, drug-eluting stents, drug-coated balloons, surgical revascularization, and intravascular brachytherapy. The aim of the current review is to describe the classification and mechanisms of ISR, provide a comprehensive and updated overview of the evidence supporting different treatment strategies, suggest a management algorithm, and present insights into future developments in the field.

## 1. Introduction

In-stent restenosis (ISR) remains the predominant cause of stent failure and the most common reason for repeat coronary revascularization [1]. Despite advances in stent technologies including thinner struts and more biocompatible polymers, as well as advancements in immunosuppressive medications, ISR still occurs at a rate of 1–2% per year [2]. Compared to patients treated for *de-novo* coronary lesions, patients with ISR have a significantly higher morbidity and mortality [3].

Understanding the mechanisms underlying ISR and providing tailored treatments based on comprehensive assessments of both patient and lesion are essential to improving clinical outcomes. The aim of this review is to delineate the classification and mechanisms of ISR as well as providing a comprehensive, and updated overview of the evidence supporting different treatment strategies and suggesting a management algorithm. Finally, we will present insights on future developments in the field.

### 1.1 Definitions

Originally, ISR was defined as a diameter stenosis of 50% or greater within the stent or within a 5 mm segment adjacent to the stent, based on angiographic visual assessment [4]. Subsequently, the Academic Research Consortium introduced a definition for clinically indicated revascularization, which also applies to ISR [5]. Under this classification, ISR is considered relevant if the stenosis diameter is at least 50% and is accompanied by signs or symptoms of ischemia, or if the stenosis degree reaches 70% or more, regardless of evidence for concomitant ischemia. Evidence of ischemia can be derived from a positive history of recurrent angina pectoris, changes on a resting electrocardiogram, or abnormal results from any non-invasive or invasive functional diagnostic test.

### 1.2 Incidence Rates

Technological improvements over the past two decades have progressively reduced the incidence of ISR [6, 7, 8]. A comprehensive meta-analysis of over 25,000 patients across 19 randomized controlled trials (RCTs) revealed a significant decline in the 1-year rate of ischemia-driven target lesion revascularization (TLR) [2]. This rate decreased from 14.7% with bare metal stent (BMS) to 4.9% and 2.5% with early generation and new-generation drug eluting stent (DES), respectively (*p*-value < 0.0001) [2]. Notably, while BMS-ISR tends to peak early after stent implantation, DES is associated with a persistent revascularization risk of approximately 2% per year [2]. Despite the declining incidence of BMS-ISR in the United States, the proportion of percutaneous coronary intervention (PCI) for ISR remains stable at around 10% per year [1]. On the other hand, the frequency of DES implantation in ISR and complexity of PCI procedures increased over time [1].

### 1.3 Clinical Presentation

ISR is commonly a gradual process that often manifests clinically as recurrent stable angina. However, in a non-negligible proportion of patients, ISR might present as acute myocardial infarction (MI) [9]. Data from the United States on patients undergoing ISR-PCI between 2009 and 2017 indicate that the most common clinical presentations were unstable angina (51.8%), followed by acute MI (27.2%), typical angina (15.1%), absence of angina symptoms (4.2%), and atypical angina (1.7%) [1]. In contrast, a large European registry revealed that only 5% of patients with ISR detected at routine control coronary angiography presented with acute coronary syndrome (ACS), while the majority (95%) experienced stable angina pectoris or were asymptomatic [10]. Of note, the occurrence of ACS in patients with ISR is associated with a higher risk of recurrent ISR and adverse cardiovascular events [10].

## 2. Risk Factors

The development of ISR is influenced by a complex interplay of patient-related, biological, vascular, procedural, and stent-related factors, as summarized in Table 1. Significant clinical risk factors for ISR include diabetes, smoking, chronic kidney disease, advanced age, and female sex [11, 12, 13]. Moreover, inflammation, drug resistance, and hypersensitivity are crucial in promoting ISR development. The implantation of foreign body elements, such as a metallic stent strut, the application of DES polymers on the coronary wall, and barotrauma lead to local inflammation characterized by leukocyte infiltration and persistent fibrin deposition. These factors hinder reendothelialization, increasing the risk of ISR and stent thrombosis [14, 15]. Genetic polymorphisms can impact the response to antiproliferative drugs, conferring resistance to sirolimus, its analogs, or paclitaxel [16]. Additionally, hypersensitivity reactions to metals like nickel and molybdenum from BMS stainless steel platforms, as well as to any of the three components of newer DES (platform, antiproliferative agent, or polymer), can potentially trigger ISR [14, 17].

**Table 1.** **Risk factors for in-stent restenosis**.

Patient related and biological factors	Anatomical factors	Procedural and stent-related factors
∙ Diabetes mellitus	∙ Long lesion length	∙ Stent underexpansion
∙ Smoking	∙ Small vessel size	∙ Stent malapposition
∙ Chronic kidney disease	∙ Severe calcifications	∙ Geographic miss
∙ Older age	∙ Tortuosity	∙ Overlapping stents
∙ Female sex	∙ High thrombus burden	∙ Small minimal lumen area post PCI
∙ Drug resistance	∙ Chronic total occlusion	∙ Stent fractures
∙ Hypersensitivity to stent materials (platform or polymer)	∙ Saphenous vein graft	∙ Thick stent struts
		∙ Use of low biocompatible polymers

PCI, percutaneous coronary intervention.

Vascular and procedural factors are probably the most significant predictors of ISR. Long lesion length, small reference vessel diameters, calcification, vessel tortuosity, high thrombus burden, and chronic total occlusion (CTO) or saphenous vein graft (SVG) as target lesions have been associated with a higher risk of restenosis (Table 1). Small vessels are more prone to ISR due to the higher risk of recoil, while heavily calcified lesions may result in stent underexpansion, one of the main predictors of ISR [18]. Procedural factors, including stent underexpansion, malapposition, longitudinal geographic miss (incomplete lesion coverage due to suboptimal stent positioning), the presence of stent gaps or overlapping stents, small minimal lumen area post PCI, and stent fractures are additional factors associated with a high risk of ISR (Table 1). Stent characteristics and designs also impact ISR risk and have been the focus of novel technological iterations. For example, newer-generation stents with thinner struts improve local blood flow dynamics, decrease shear stress, and improve both stent coverage and healing, potentially reducing the need for repeat revascularization and improving clinical outcomes [19]. Innovations such as bioresorbable polymer or polymer free technologies are also being explored to decrease inflammation and the onset of neoatherosclerosis associated with DES that contain permanent polymers [6, 7].

## 3. Mechanisms and Morphological Patterns of ISR

The morphological appearance of ISR, observable through histologic examination or intravascular imaging techniques, can be classified into mechanical or biological patterns. Often, these patterns demonstrate considerable overlap.

### 3.1 Mechanical Patterns

**Fig. 1.**
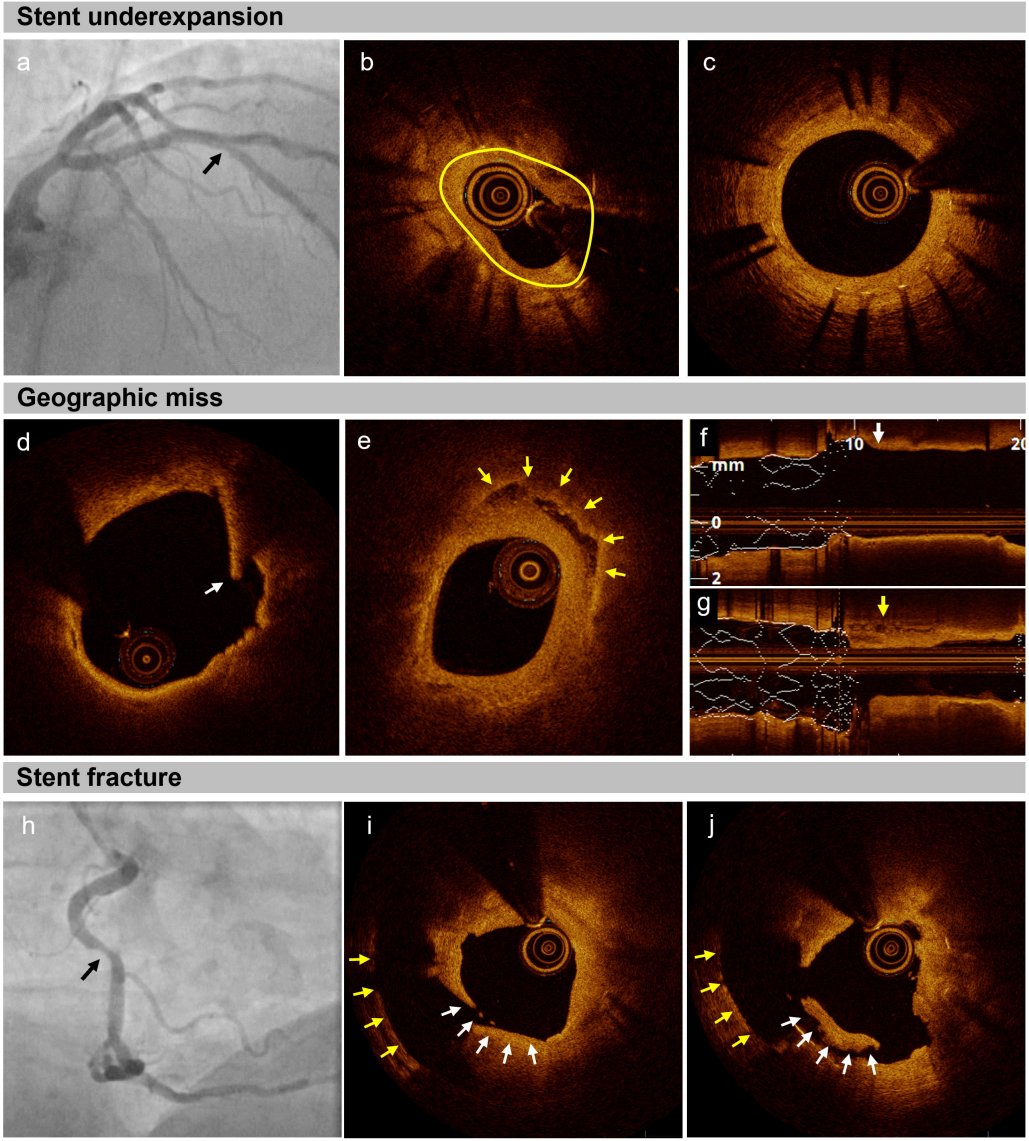
**Mechanical patterns of in-stent restenosis (ISR) in optical coherence tomography (OCT)**. (a–c) Stent underexpansion. (a) Coronary angiography displaying ISR in the mid left anterior descending artery (black arrow). (b) The reduced stent area (2.79 mm^2^, yellow line) resulted from absolute stent underexpansion. (c) The expansion of the distal portion of the stent was adequate. (d–g) Geographic miss: a drug-eluting stent (DES) was implanted in a patient with acute myocardial infarction. (d,f) During the index procedure, a thin cap fibroatheroma with intimal disruption (white arrows) was detected within 5 mm proximal to the implanted DES. (e,g) At the three-year follow-up, OCT imaging showed ISR, characterized by the presence of a layered plaque (yellow arrows) and lumen narrowing. (h–j) Stent fracture. (h) The coronary angiograph shows an ISR in the mid right coronary artery (black arrow). (i,j) The OCT image demonstrates an abrupt change of the stent strut position (white arrows) between 2 frames (within 0.4 mm), with the yellow arrows indicating the vessel wall.

Stent underexpansion during the index PCI is one of the most common causes of ISR [20, 21], serving as an independent predictor of both TLR and major adverse cardiac events [22, 23]. The determinants of stent underexpansion include both lesion characteristics and procedural issues. Lesion-specific factors include long, circumferential, and deep calcifications, while procedural issues encompass suboptimal lesion preparation or stent undersizing (Fig. 1).

Stent malapposition, which refers to the absence of contact between stent struts and the vessel wall, can occur either during the index PCI or develop months or years later due to positive remodeling of the vessel [24, 25]. Although acute malapposition is observed in over 70% of patients according to optical coherence tomography (OCT) studies, its causal association with stent failure remains unclear [24, 26]. Small malapposition (less than 400 µm with a longitudinal extension below 1 mm) typically result in the incorporation of the stent struts into the vessel wall, whereas larger stent malappositions (above 400 µm and/or longitudinal extensions longer than 1 mm) are associated with an increased risk of thrombus formation [25, 27]. Employing balloon pre-dilation and ensuring optimal stent apposition through balloon post-dilation are crucial strategies to reduce the risk of stent failure and ensure uniform anti-proliferative drug delivery across the vessel walls [23].

Geographic misses and residual plaque burden at the stent’s landing zone increase the risk of edge restenosis, leading to significantly higher rates of repeat revascularization even at short-term follow-ups [28] (Fig. 1). Additionally, residual stent edge dissection can induce neoatherosclerosis due to the resultant arterial wall damage. Stent edge dissections detected by intravascular ultrasound (IVUS), represent significant independent predictors of TLR within one year [29]. However, the prognostic impact of residual non-flow-limiting dissections remains contentious, as partial or complete healing frequently occur over time [25, 30, 31].

Moreover, stent fractures are a relatively common cause of ISR and tend to occur in longer or tortuous lesions [32, 33] (Fig. 1). The absence of metallic coverage at the fracture site, coupled with local irritation from fractured struts and compromised drug delivery, promote early neointimal proliferation [34].

### 3.2 Biological Patterns

**Fig. 2.**
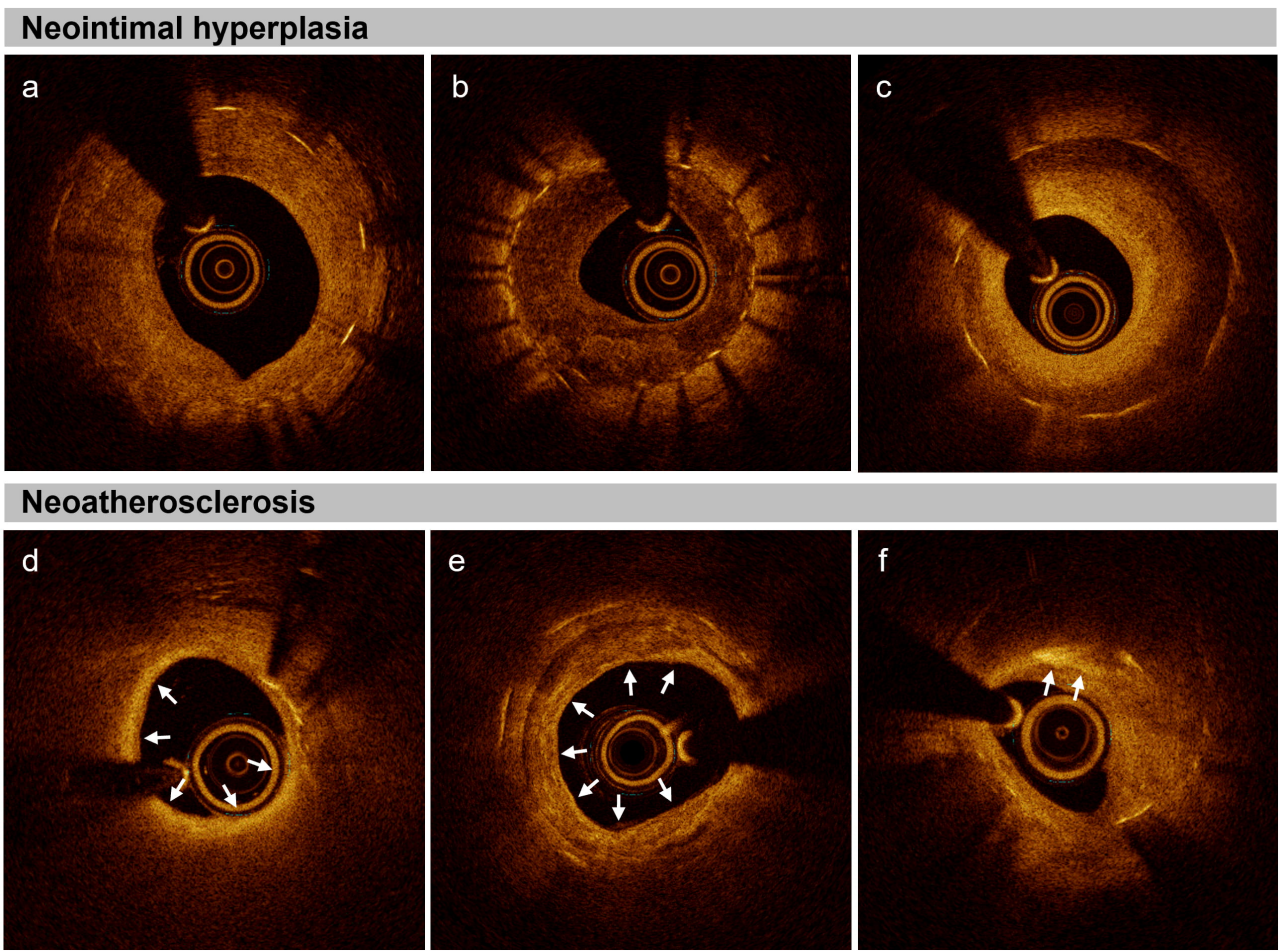
**Neointimal hyperplasia and neoatherosclerosis in optical coherence tomography**. (a) Homogenous neointima characterized by a uniformly signal-rich appearance, indicative of a dense, uniform cellular structure. (b) Heterogenous neointima, characterized by a signal-poor appearance with areas of varying signal intensity, suggesting variations in cellular and extracellular matrix composition. (c) Layered neointima, marked by a signal-poor base with a high-signal band adjacent to the luminal surface, representing layered cellular growth. (d) Neoatherosclerosis with in-stent fibroatheroma, presenting as a signal-poor region with high attenuation and diffuse borders indicating lipid accumulations (white arrows). (e) Neoatherosclerosis with in-stent calcification, characterized by signal-poor regions with low attenuation and distinct borders indicating areas of calcification (white arrows). (f) Neoatherosclerosis with in-stent cholesterol crystals, characterized as thin and linear structures within the neointima, exhibiting high backscattering without attenuation, indicative of cholesterol crystal formation (white arrows).

ISR not induced by mechanical issues is primarily driven by two mechanisms: neointimal hyperplasia (NIH) and neoatherosclerosis [18] (Fig. 2). NIH involves excessive proliferation of smooth muscle cells and the accumulation of extracellular matrix, which are intensified vessel’s healing responses stimulated by stent implantation [11]. In BMS, this response often manifests as “hypercellular NIH”, which is characterized by a higher concentration of smooth muscle cells and a moderate amount of proteoglycan [12, 35]. In contrast, DES-ISR exhibits a “hypocellular” pattern due to reduced endothelial injury and the elution of antiproliferative agents [36]. Although NIH is a common pattern of ISR in BMS, it can also be observed in first-generation DES [37].

Neoatherosclerosis is one of the most common mechanisms contributing to ISR and stent thrombosis in patients undergoing implantation of newer generation DES [12, 38, 39, 40, 41]. Distinct from NIH, neoatherosclerosis involves the development of atherosclerotic changes within the stented segment, which can range from simple intimal thickening to high-risk patterns like fibro-calcific plaque or thrombosis-prone thin-cap fibroatheroma [12, 40]. Lipidic neoatherosclerosis, compared to calcified neoatherosclerosis or no atherosclerosis, has emerged as the main determinant of repeat revascularizations [38].

## 4. Intracoronary Imaging for In-Stent Restenosis

### 4.1 Angiography

Coronary angiography remains the first-line diagnostic tool for ISR, facilitating the assessment of its geographical distribution and severity according to Mehran’s classification (Table 2, Ref. [4, 18, 42, 43, 44]) [4]. With the aid of stent-boost technologies, angiography can identify stent fracture, geographic miss, and edge dissection, obviating the need for intracoronary imaging in selected cases [45, 46].

**Table 2.** **Classifications of in-stent restenosis**.

Classification	Required tools	Classes	Description - subclassification
Mehran *et al*., 1999 [4]	Angiography	I: Focal	Lesion with length ≤10 mm and located at:
			∙ Type IA: unscaffolded segment (i.e., articulation or gap)
			∙ Type IB: stent body (type IB)
			∙ Type IC: proximal or distal margin
			∙ Type ID: a combination of these sites (multifocal ISR)
		II: Diffuse	Lesion(s) with length >10 mm:
			∙ confined to the stent body
			∙ not extended beyond stent margins
		III: Diffuse proliferative	Lesion(s) with length >10 mm:
			∙ extended beyond stent margins
		IV: Total occlusion	Lesion(s) with TIMI flow grade of 0
Kang* et al*., 2010 [42]	IVUS	Focal	MLA <4 mm^2^ and lesion length ≤10 mm and:
			∙ confined to the stent body (focal body sub-type)
			∙ extended to stent margins (focal marginal sub-type)
		Multifocal	Multiple focal lesions:
			∙ confined to the stent body (multifocal body sub-type)
			∙ involving stent margins (multifocal marginal sub-type)
		Diffuse	MLA <4 mm^2^ and lesion length >10 mm:
			∙ confined to stent body (diffuse body sub-type)
			∙ extending to stent margins (diffuse marginal sub-type)
Ali *et al*., 2013 [43] (classification of neoatherosclerosis)	OCT	I: Thin-cap fibroatheroma	Area of thin cap (<65 µm), signal-poor region with diffuse borders located between the lumen and the stent struts
		II: Thick-cap fibroatheroma	No area of thin cap, signal-poor region with diffuse borders located between the lumen and the stent struts
		III: Peristrut neoatheroma	Signal-poor region with diffuse borders located around the stent struts
		IV: Preexisting fibroatheroma	Signal-poor region with diffuse borders located between the stent struts and the adventitia
Shlofmitz *et al*., 2019 [18]	Angiography and OCT	I: Mechanical pattern	∙ Type IA: Underexpansion or undersizing
			∙ Type IB: Stent fracture
		II: Biological pattern	∙ Type IIA: Neointimal hyperplasia, homogeneous bright layer with few or no backscattering
			∙ Type IIB: Non-calcified neoatherosclerosis
			∙ Type IIC: Calcified neoatherosclerosis
		III: Mixed pattern	Combination of biological and mechanical factors
		IV	Chronic total occlusion
		V	≥2 layers of stent
SCAI Classification, 2023 [44]	Angiography, OCT, and clinical history	Early: <30 days	Undersizing, underexpansion, or stent fracture
		Late: 30 days – 1 year	Delayed healing, uncovered stent struts, or neointimal hyperplasia
		Very late: >1 year	Neoatherosclerosis, neointimal hyperplasia, or stent fracture

ISR, in-stent restenosis; IVUS, intravascular ultrasound; MLA, minimum lumen area; OCT, optical coherence tomography; SCAI, Society for Cardiovascular Angiography and Interventions, TIMI, thrombolysis in myocardial infarction.

### 4.2 Intravascular Ultrasound

Due to its high spatial penetration, IVUS is instrumental in characterizing certain morphological patterns of ISR, measuring the vessel lumen, and detecting stent underexpansion or undersizing. Currently accepted cut-offs for optimal stent expansion include a minimum stent area (MSA) greater than 5.5 mm^2^ (or over 8–8.5 mm^2^ for left main PCI) [20, 21] and a relative stent expansion (MSA to distal vessel reference area ratio) of at least 80% and ideally exceeding 90% [25]. However, IVUS exhibits lower spatial resolution compared to OCT, which limits its ability to accurately discern other ISR patterns and precludes the evaluation of tissue composition [25].

### 4.3 Optical Coherence Tomography

To date OCT stands as the most precise intravascular imaging tool for detecting lumen and stent-related morphologies, as well as for assessing the mechanisms of stent failure (Figs. 1,2) [47]. The superior resolution of OCT allows for the visualization of individual stent struts, detection of stent fractures, strut malappositions, and edge dissections, which may go unnoticed with angiography and IVUS [30]. In addition, OCT provides detailed insights into the tissue composition of ISR by analyzing intraluminal material, lumen contour, variations in backscattering patterns, and the presence of microvessels [48]. Of note, MSA often appears smaller when assessed by OCT than by IVUS; consequently, the recommended MSA cut-off for an optimal PCI result is lower for OCT (set above 4.5 mm^2^) [25].

### 4.4 Prognostic Benefit and Guideline Recommendations

The prognostic benefits of routine intracoronary imaging for ISR treatment remain uncertain due to the absence of dedicated and adequately powered RCTs [49, 50]. However, a recent meta-analysis of RCTs demonstrated that PCI of *de-novo* lesions, when guided by intravascular imaging techniques with either IVUS or OCT, significantly enhances both the safety and effectiveness of the procedure and is associated with a lower risk of restenosis compared to PCI guided solely by angiography [51]. Since IVUS and OCT allow for full characterization of the mechanisms of stent failure, their routine use for ISR management is recommended with a class IIa indication by the latest myocardial revascularization guidelines [52, 53].

## 5. Classification of ISR

Historically, ISR has been categorized as either focal (≤10 mm in length) or diffuse (>10 mm). In 1999, Mehran *et al*. [4] expanded this classification to include a four-class scheme that added diffuse proliferative and occlusive patterns to the original two categories (Table 2). This scheme also considered the NIH location relative to the implanted stent (i.e., body, margin, outside the stent). The prognostic value of Mehran’s classification was validated for both BMS- and DES-ISR, with a progressive increase in TLR from class I to class IV ISR [4, 54, 55]. This classification is frequently used to describe ISR in RCTs.

The American College of Cardiology/American Heart Association (ACC/AHA) lesion classification, originally developed for de novo coronary disease, has been validated for ISR, demonstrating a significant correlation between type B2 or C lesions with worse acute outcomes and an increased restenosis rate over time [56]. In the intravascular imaging era, Kang *et al*. [42] proposed an IVUS-based classification for ISR, accounting for minimum lumen area, lesion length, and the localization of restenosis in relation to the stent. Additionally, Ali *et al*. [43] and Gonzalo *et al*. [48] introduced ISR classifications based on OCT findings. The only classification specifically designed to guide clinical treatment was proposed by Shlofmitz and Waksman *et al*. [18], which categorizes ISR into mechanical and biological patterns based on angiographic and OCT assessment. Finally, the SCAI introduced a classification of ISR comprising three categories according to the time elapsed between the index PCI and the onset of ISR [44].

## 6. Treatment

### 6.1 Ballon Angioplasty

Balloon angioplasty (BA) was the earliest treatment modality developed for ISR. This technique is technically straightforward and often yields favorable immediate angiographic results through longitudinal and axial tissue extrusion and stent expansion. However, the long-term outcomes of BA are less satisfactory. Management with BA alone is associated with ISR recurrence rates up to 50%, largely due to severe tissue proliferation [57, 58]. Furthermore, studies have demonstrated that BA is inferior to newer treatment modalities (Table 3, Ref. [59, 60, 61, 62, 63, 64, 65, 66, 67, 68, 69, 70, 71, 72, 73, 74, 75, 76, 77, 78, 79, 80, 81, 82, 83, 84, 85, 86, 87, 88, 89, 90, 91, 92, 93, 94, 95, 96, 97, 98, 99, 100, 101, 102, 103, 104, 105, 106, 107, 108, 109, 110, 111, 112]) [113, 114]. Therefore, BA in isolation is not recommended except for specific ISR patterns. BA should be used to prepare the lesion prior to other adjunctive therapies or final treatment with DES or drug-coated balloons (DCB) (Table 4, Ref. [52, 53, 57, 58, 59, 60, 61, 62, 63, 64, 65, 66, 67, 68, 69, 70, 71, 72, 73, 74, 75, 76, 77, 78, 79, 80, 81, 82, 83, 84, 85, 86, 87, 88, 89, 113, 114, 115, 116, 117, 118, 119, 120, 121, 122, 123, 124, 125, 126, 127, 128, 129, 130, 131, 132, 133, 134, 135, 136, 137, 138, 139]). The use of longer balloons or those with non-slip elements can mitigate the risk of stent edge injuries caused by balloon slippage, known as the “watermelon seeding” phenomenon [59, 115]. Despite its limitations as a standalone treatment, BA, especially using ultra-high-pressure noncompliant (UHPNC) balloons as sole treatment modality, retains an important role in cases of stent underexpansion [116]. To minimize the risk of vessel perforation, downsizing UHPNC balloons is a recommended practice.

**Table 3.** **Randomized controlled trials on devices for the treatment of in-stent restenosis**.

	Trial name, year	Sample size	Stent type	Treatments	Primary endpoint	Follow up	Results	Main finding
IVBT	Teirstein *et al*., 1997 [87]	55	BMS	IVBT vs placebo	LLL and late loss index	6	LLL: 0.38 ± 1.06 vs 1.03 ± 0.97, *p* = 0.03; late loss index: 0.12 ± 0.63 vs 0.60 ± 0.43, *p* < 0.01	IVBT better
	Waksman *et al*., 2000 [89]	130	BMS	IVBT vs placebo	Death, MI, TLR	6	29.2% vs 67.6%, *p* < 0.001	IVBT better
	Schühlen *et al*., 2001 [90]	21	BMS	IVBT vs no treatment	LLL	6	0.81 ± 0.93 vs 1.91 ± 0.41, *p* = 0.003	IVBT better
	GAMMA-1, 2001 [84]	252	BMS	IVBT vs placebo	MACE*	9	28.2% vs 43.8%, *p* = 0.02	IVBT superior
	INHIBIT, 2002 [88]	332	BMS	IVBT vs placebo	Death, MI, TLR; Binary stenosis	9	15% vs 31%, *p* = 0.0006	IVBT superior
							26% vs 52%, *p* < 0.0001	
	START, 2002 [85]	476	BMS	IVBT vs placebo	TVR	8	16.5% vs 26.2%, *p* = 0.012	IVBT superior
	Reynen *et al*., 2006 [86]	165	BMS	IVBT vs placebo	Restenosis rate^#^	6	24% vs 40%, *p* = 0.04	IVBT superior
CB	RESCUT, 2004 [60]	428	BMS	CB vs BA	≥50% restenosis	7	29.8% vs 31.4%, *p* = 0.82	No difference (CB not superior)
	Montorsi *et al*., 2004 [61]	50	BMS	CB vs BA	MACE**	6 ± 1	MACE: 17% vs 28%, *p*-value ns	CB less TLR
					TLR		TLR: 12.5% vs 40%, *p* < 0.05	
SB	PATENT-C, 2016 [63]	61	BMS	paclitaxel coated SB vs SB	LLL	6	0.17 ± 0.40 vs 0.48 ± 0.51, *p* = 0.01	Paclitaxel-coated SB superior
	ISAR-DESIRE 4, 2017 [62]	252	DES	SB vs standard therapy before DCB	In-segment % diameter stenosis	6–8	35.0 ± 16.8% vs 40.4 ± 21.4%, *p* = 0.047	SB superior
HPNCB	ELEGANT, 2019 [59]	105	BMS & DES	BA with NSE vs HPNCB before DCB	LLL	8	0.28 ± 0.45 vs 0.27 ± 0.38, *p* = 0.75	No difference (BA with NSE not superior)
ELCA	Haase *et al*., 1999 [91]	96	BMS	ELCA+BA vs BA	Clinical endpoint°	3–6	26% vs 31%, *p* = ns	No difference
	Sato *et al*., 2020 [66]	40	BMS & DES	ELCA+DCB vs DCB	Clinical endpoint^†^	12	No significant difference (rates not reported)	No difference
RA	ARTIST, 2002 [65]	298	BMS	RA+BA vs BA alone	Net gain MLD	6	0.45 ± 0.57 vs 0.67 ± 0.54, *p* = 0.0019	RA+BA superior
	ROSTER, 2004 [64]	200	BMS	RA+BA vs BA alone	TLR	9	32% vs 45%, relative reduction 32%, *p* = 0.042	RA+BA superior
	Ragosta *et al*., 2004 [92]	113	BMS	RA vs BMS (diffuse lesions)	Cardiac death, MI and TLR	9	43% vs 32%	No difference
BMS	RIBS, 2003 [67]	450	BMS	BMS vs BA	Binary restenosis	6	33% vs 38%, *p* = 0.36	No difference
	Ragosta *et al*., 2004 [92]	113	BMS	BMS vs BA (focal lesions)	Cardiac death, MI and TLR	9	7% vs 21%	No difference
	Alfonso *et al*., 2005 [93]	40	BMS	BA+BMS vs BA alone	Early lumen loss 30–60’ after PCI	-	0.0025 ± 0.4 vs 0.92 ± 0.9, *p* = 0.0001	BA+BMS better
Drug-eluting stents	**DES vs BA**							
	ISAR DESIRE, 2005 [69]	300	BMS	SES vs PES vs BA	Binary restenosis	6	SES 14.3% vs BA 44.6%, *p* < 0.001	SES and PES superior to BA
							PES 21.7% vs BA 44.6%, *p* < 0.001	
	RIBS II, 2006 [94]	150	BMS	SES vs BA	Binary restenosis	9	11% vs 39%, *p* < 0.001	SES superior
	CRISTAL, 2012 [68]	281	DES	SES vs BA	LLL	9–12	Among 197 DES-ISR: 0.37 ± 0.57 vs 0.41 ± 0.63, *p* = 0.73	No difference (SES not superior)
	Song *et al*., 2012 [95]	96+66	DES	SES vs CB	LLL (analysis segment)	9	SES vs CB: 0.06 vs 0.25, *p* = 0.04	SES superior to CB no diff SES/EES
				SES vs EES			SES vs EES: 0.11 vs 0.00, *p* = 0.64	
	**DES vs IVBT**							
	INDEED, 2008 [70]	129	BMS	SES vs IVBT	Segment late loss	6	Target segment: 0.23 ± 0.59 vs 0.40 ± 0.72, *p* = 0.199; analysis segment: 0.15 ± 0.62 vs 0.55 ± 0.69, *p* = 0.003	SES superior
	Wiemer *et al*., 2011 [71]	91	Not reported	SES vs IVBT	LLL	6	0.09 ± 0.29 vs 0.39 ± 0.79, *p* = 0.020	SES superior
	**DES vs DES**							
	ISAR-DESIRE 2, 2010 [72]	450	DES	SES vs PES	LLL (in-segment)	6–8	0.40 ± 0.65 vs 0.38 ± 0.59, *p* = 0.85	No difference (SES not superior)
	RESTENT-ISR, 2016 [73]	304	DES	EES vs ZES	Neointima volume	9	0.51 ± 0.48 vs 0.56 ± 0.54, *p* = 0.47	No difference (EES not superior)
Drug-eluting balloons	**PCB vs BA**							
	PACCOCATH ISR, 2006 [78]	52	BMS	PCB vs BA	LLL	6	0.03 ± 0.48 vs 0.74 ± 0.86, *p* = 0.002	PCB superior
	Habara *et al*., 2011 [75]	50	SES	PCB vs BA	LLL (in-segment)	6	0.18 ± 0.45 vs 0.72 ± 0.55, *p* = 0.001	PCB better
	PACCOCATH ISR I/II, 2012 [77]	108	BMS 96%	PCB vs BA	LLL (in-segment)	6	0.11 ± 0.44 vs 0.80 ± 0.79, *p* = 0.001	PCB better
							Diff 0.69 (95% CI 0.44–0.96)	
	PEPCAD DES, 2012 [76]	110	DES	PCB vs BA	LLL (target lesion)	6	0.43 ± 0.61 vs 1.03 ± 0.77, *p* < 0.001	PCB superior
	Habara *et al*., 2013 [74]	208	BMS 58%	PCB vs BA	Cardiac death, MI or TVR	6	6.6% vs 31%, *p* < 0.001	PCB superior
	AGENT IDE, 2024 [79]	600	DES 90%	PCB vs BA	Cardiac death, TV-MI, ischemia driven TLR	12	17.9% vs 28.6%, *p* = 0.03	PCB superior
							HR 0.59 (95% CI 0.42–0.84)	
	**PCB vs DES**							
	PEPCAD II, 2009 [96]	131	BMS	PCB vs PES	LLL (in segment)	6	0.17 ± 0.42 vs 0.38 ± 0.61, *p* = 0.03	PCB superior
							Diff –0.21 (–0.40 to –0.02)	
	ISAR DESIRE 3, 2013 [97]	402	DES	PCB vs PES vs BA	Diameter stenosis	6–8	PCB vs PES: 38 ± 21.5% 37.4 ± 21.8%, *p* (non-inf) = 0.007	PCB noninferior to PES
							BA: 54.1 ± 25.0%, *p* (sup) < 0.0001	PCB and PES superior to BA
	SEDUCE, 2014 [98]	50	BMS	PCB vs EES	% uncovered struts (OCT)	9	1.4 ± 1.9% vs 3.2 ± 3.4%, *p* = 0.025	PCB better
	PEPCAD China ISR, 2014 [99]	220	DES	PCB vs EES	LLL (in segment)	9	0.46 ± 0.51 vs 0.55 ± 0.61	PCB noninferior
							*p* (non-inferiority) = 0.0005	
	RIBS V, 2014 [100]	189	BMS	EES vs PCB	MLD (in segment)	9	2.36 ± 0.6 vs 2.0 ± 0.6, *p* < 0.001	EES superior
	TIS, 2016 [101]	309	BMS	PCB vs EES	LLL (in segment)	12	0.09 ± 0.44 vs 0.44 ± 0.73, *p* = 0.0004	PCB superior
							Diff 0.354 (95% CI 0.149–0.558)	
	DARE, 2018 [102]	278	DES	PCB vs EES	MLD (in segment)	6	1.71 ± 0.51 vs 1.74 ± 0.61	PCB noninferior
							*p* (noninferiority) < 0.0001	
	BIOLUX, 2018 [103]	229	DES	PCB vs BP-SES	LLL (in-stent)	6	0.03 ± 0.4 vs 0.20 ± 0.7 *p* = 0.398	PCB noninferior
							*p* (noninferiority) < 0.0001	
	RESTORE, 2018 [104]	172	DES	PCB vs EES	LLL (target segment)	9	0.15 ± 0.49 vs 0.19 ± 0.41, *p* = 0.54	No differences (EES not superior)
	**PCB vs PCB**							
	Agent ISR, 2020 [105]	125	BMS/DES	PCB vs PCB	LLL (in stent)	6	0.397 ± 0.43 vs 0.939 ± 0.536	PCB Agent noninferior
				Agent vs SeQuent			*p* (non-inferiority) = 0.046	
	RESTORE ISR China, 2018 [106]	240	DES	PCB vs PCB	LLL (in segment)	9	0.38 ± 0.50 vs 0.35 ± 0.47	PCB Restore noninferior
				Restore vs SeQuent			*p* (noninferiority) = 0.046	
	Zhu *et al*., 2021 [107]	216	DES	PCB vs PCB	In segment late loss	9	0.29 ± 0.43 vs 0.30 ± 0.46	PCB Shenqui noninferior
				Shenqui vs SeQuent			*p* (noninferiority) = 0.002	
	Hu *et al*., 2021 [108]	211	BMS/DES	PCB vs PCB	LLL (target lesion)	9	0.35 ± 0.42 vs 0.38 ± 0.45	PCB LONGTY noninferior
				Longty vs Sequent			*p* (noninferiority) < 0.001	
	Jun et al., 2022 [109]	82	DES 88%	PCB vs PCB	LLL (in-segment)	6	0.15 ± 0.43 vs 0.24 ± 0.39	PCB Genoss noninferior
				Genoss vs Sequent			*p* (noninferiority) = 0.001	
	DISSOLVE A, 2023 [110]	260	DES	PCB vs PCB	In segment late loss	9	0.50 ± 0.06 vs 0.47 ± 0.07	PCB Dissolve noninferior
				Dissolve vs SeQuent			p (noninferiority) = 0.03	
	**SCB vs PCB**							
	Ali *et al*., 2019 [80]	50	DES	SCB vs PCB	LLL	6	0.18 ± 0.54 vs 0.31 ± 0.62, p = ns	No difference
				SeQuent vs SeQuent				
	Scheller *et al*. 2022 [83]	101	DES	SCB vs PCB	LLL (in-segment)	6	0.26 ± 0.60 vs 0.25 ± 0.57	SCB noninferior
				SeQuent vs SeQuent			Noninferiority met	
	TIS-2, 2023 [111]	128	BMS/DES	SCB vs PCB	LLL (in-segment)	12	LLL difference: –0.277 to 0.229	SCB not noninferior
				MagicTouch vs SeQuent			Noninferiority of SCB not met	
	Chen *et al*., 2023 [81]	258	DES	SCB vs PCB	LLL (stented segment)	9	0.35 ± 0.47 vs 0.31 ± 0.36	SCB noninferior
				SeQuent vs SeQuent			*p* = 0.82	
	**BCB vs PCB**							
	REFORM, 2023 [112]	201	Not reported	BCB vs PCB	In-segment diameter stenosis	6	41.8% vs 31.2%	BCB not noninferior
				Biosensors vs SeQuent			Non inferiority of BCB not met	
	BIO ASCEND ISR, 2024 [82]	280	DES	BCB vs PCB	LLL (in-segment)	9	0.23 ± 037 vs 0.25 ± 0.35	BCB noninferior
				BioAscend vs SeQuent			*p* (non-inferiority) < 0.0001	

* Composite of: death, MI, emergency bypass surgery, TLR. ** Composite of: death, Q/non-Q wave MI, repeat PCI or coronary artery bypass surgery. # Restenosis >50% and anginal symptoms or new myocardial ischemia in stress test. ° Primary clinical endpoint: composite of death, MI, bypass surgery, or a second PTCA involving the previously treated in-stent. ^†^ Composite of death from any causes, cardiac death, MI, and stent thrombosis. Abbreviations: BA, balloon angioplasty; BCB, biolimus-coated balloon; BMS, bare metal stent; BP-SES, biodegradable polymer sirolimus-eluting stent; CB, cutting balloon; DCB, drug-coated balloon; DES, drug eluting stent; EES, everolimus eluting stent; ELCA, excimer laser coronary angioplasty; HPNCB, high-pressure non-compliant balloon; ISR, in-stent restenosis; IVBT, intravascular brachytherapy; LLL, late lumen loss; MDS, mean diameter stenosis; MACE, major adverse clinical events; MI, myocardial infarction; MLD, minimal lumen diameter; ns, non-significant; NSE, non-slip element; OCT, optical coherence tomography; PCB, paclitaxel-coated balloon; PCI, percutaneous coronary intervention; PES, paclitaxel eluting stent; RA, rotational atherectomy; SB, scoring balloon; SCB, sirolimus coated balloon; SES, sirolimus eluting stent; TLR, target lesion revascularization; TV-MI, target vessel myocardial infarction; TVR, target vessel revascularization; ZES, zotarolimus eluting stent.

**Table 4.** **Advantages, drawbacks and recommended use of treatment modalities for ISR**.

Treatment modality	Advantages	Drawbacks	Recommended use
Ballon angioplasty [57, 58, 59, 113, 114, 115, 116]	∙ Technically simple ∙ Good immediate angiographic results	∙ Risk of edge dissections ∙ Very high rates of restenosis if used as standalone treatment	∙ Lesion preparation ∙ Stent underexpansion (as standalone treatment) ∙ Longer balloons or balloons with non-slip elements to avoid slippage ∙ UHPNC ballons if resistant stent underexpansion
Cutting and scoring balloons [60, 61, 62, 63, 117]	∙ Reduce the number of balloons needed for lesion preparation	∙ Very high rates of restenosis if used as standalone treatment	∙ Resistant stent underexpansion due to calcification or significant neointimal hyperplasia
	∙ Reduce ballon slippage		
	∙ Modify neointimal growth and ↑ antiproliferative drug delivery		
Intravascular lithotripsy [57, 118, 119, 120, 121]	∙ Induces fractures of vascular calcium ∙ Compared with atheroablative therapies: lower risk of perforation; less dependent on operator experience	∙ Evidence limited to observational studies ∙ Not as standalone treatment	∙ Calcified lesions with resistant stent underexpansion
Rotational atherectomy [64, 65, 122, 123]	∙ Physical removal of neointimal, neoatherosclerotic and calcified tissue	∙ Not negligeable risk of vessel perforation and burr entrapment	∙ Resistant stent underexpansion due to calcification or significant neointimal hyperplasia
Orbital atherectomy [124, 125, 126]	∙ Physical removal of neointimal, neoatherosclerotic and calcified tissue ∙ Compared to RA: better for large diameter vessels and allow bidirectional atheroablation	∙ Off-label for the treatment of ISR ∙ Risk of vessel perforation and burr entrapment	∙ Resistant stent underexpansion due to calcification or significant neointimal hyperplasia in large diameter lesions
Excimer Laser Coronary Angioplasty [66, 127, 128, 129]	∙ Physical removal of neointimal, neoatherosclerotic and calcified tissue ∙ Risk of vessel perforation and no-reflow phenomenon	∙ Limited evidence in the setting of ISR	∙ Resistant stent underexpansion due to calcification or significant neointimal hyperplasia
Repeat DES implantation [52, 53, 67, 68, 69, 70, 71, 72, 73, 113, 114, 130, 131, 132, 133, 134, 135]	∙ Strategy associated with the best short and long-term outcomes	∙ Requires DAPT or more potent antiplatelet regimen for a longer time compared to DCB	∙ Treatment of choice for ISR, except when the mechanism is stent underexpansion
		∙ Not recommended in case of multilayer ISR	∙ Particularly indicated in case of stent fracture or stent gap
DCB [52, 53, 74, 75, 76, 77, 78, 79, 80, 81, 82, 83, 113, 114, 136, 137]	∙ Avoid an additional stent layer ∙ Requires DAPT or more potent antiplatelet regimen for a shorter time compared to DES	∙ Not optimal in case of suboptimal lesion expansion after BA and ISR with aggressive patterns (diffuse or occlusive)	∙ Multilayer ISR ∙ ISR of BMS ∙ Focal ISR ∙Patients at high bleeding risk
Intravascular brachytherapy [52, 84, 85, 86, 87, 88, 89, 138, 139]	∙ Inhibits neointimal growth with intracoronary delivery of beta radiation	∙ Issues related with radioprotection/radiation dosing	∙ Recurrent ISR with multiple stent layers and failed treatment with DCB
		∙ Need for prolonged antiplatelet therapy due to delayed endothelialization	
CABG [52, 53, 135]	∙ Optimal long-term results especially if arterial graft is used ∙ Only applicable if the coronary segment distally of the ISR is suitable for graft anastomosis	∙ Not applicable in patients at high surgical risk	∙ Recurrent ISR with multiple stent layers and failed treatment with DCB

BA, balloon angioplasty; BMS, bare metal stent; CABG, coronary artery bypass graft; DCB, drug coated balloon; DAPT, dual antiplatelet therapy; DES, drug eluting stent; ISR, in-stent restenosis; RA, rotational atherectomy; UHPNC, ultra-high-pressure noncompliant.

### 6.2 Cutting and Scoring Balloons

Cutting balloons (CB) feature a standard angioplasty balloon equipped with three to four longitudinal metallic microtomes that incise the atherosclerotic plaque upon ballon inflation. Compared to BA, CBs achieve greater lumen gains and exhibit reduced elastic recoil in *de-novo* lesions [117]. In the context of BMS-ISR, using CB (compared to BA) was associated with the use of fewer balloons for lesion preparation and less balloon slippage, but similar rates of angiographic restenosis at 7 months [60]. In contrast, a small RCT reported a reduction in TLR at 6 months with CB usage [61]. To date, no RCTs have evaluated the safety and efficacy of CB in DES-ISR.

Scoring balloons (SB) consist of a semicompliant balloon surrounded by a nitinol-based scoring edge arranged in a spiral formation, which creates discrete incisions in coronary artery walls. The ISARDESIRE 4 (Intracoronary Stenting and Angiographic Results: Optimizing Treatment of Drug-Eluting Stent In-Stent Restenosis 4) trial demonstrated that using SB instead of traditional BA prior to DCB was associated with lower diameter stenosis at 6 months after PCI of DES-ISR; however, the trial was underpowered to assess differences in clinical outcomes [62] (Table 3). The incisions made by the SB potentially enhance the delivery and retention of the antiproliferative agent from the DCB. A device combining the features of SB and DCB (a paclitaxel-coated SB) yielded superior angiographic results compared to an uncoated SB in the treatment of BMS-ISR [63].

In summary, CB and SB are valuable options for lesion preparation prior to definitive treatment with DES or DCB, particularly in undilatable or calcified ISR (Table 4). Both SB or CB facilitate higher lumen gains, improved drug delivery, and reduced the risk of balloon slippage when compared to BA. Conversely, the use of CB or SB as standalone treatment is not recommended due to their inability to inhibit neointimal proliferation, leading to high rates of recurrent stenosis comparable to those observed with BA alone.

### 6.3 Intravascular Lithotripsy

Intravascular lithotripsy (IVL), specifically the C2 Shockwave Medical Coronary IVL system (Shockwave Medical, Santa Clara, CA, USA), is a novel technology that integrates multiple lithotripsy emitters at the tip of a balloon-like platform [118]. This device generates sonic pressure waves that fracture vascular calcium and increase the compliance of calcified coronary plaques [118]. However, the technology’s efficacy may be somewhat diminished by the presence of metallic struts and neointimal layers. Additionally, there is a risk that IVL could disrupt recently implanted DES polymers, compromising the delivery of the antiproliferative agent. Currently, evidence supporting IVL use for ISR treatment is limited to observational studies [119, 120, 121]. Despite these challenges, IVL presents significant benefits for managing calcified and undilatable ISR. Compared with atheroablative devices, IVL has a lower risk of vessel perforation, is more user-friendly, and is less dependent on operator experience [57] (Table 4).

### 6.4 Atheroablative Therapy

Atheroablative devices were developed to physically remove neointimal, neoatherosclerotic, and calcified tissues to improve luminal gain.

Rotational atherectomy (RA) utilizes a diamond-encrusted elliptical burr rotating at high speeds (140,000 to 180,000 rpm) attached to a drive shaft, which advances gradually across the lesion over a guidewire [122]. The use of RA for BMS-ISR treatment has demonstrated lower rates of TLR at 9 months in a small single-center RCT [64], but resulted in higher rates of lumen restenosis and procedural complications in a larger multicentric trial [65] (Table 3). Presently, there is no randomized data supporting the use of RA for the treatment of DES-ISR. Based on this evidence, the routine use of RA for ISR treatment is not recommended (Table 4). Nevertheless, RA may be beneficial in specific cases of calcified ISR with resistant stent underexpansion, as suggested by some case reports [123].

The orbital atherectomy (OA) system utilizes a diamond-coated crown mounted on a sheath-covered drive shaft. This assembly advances over a dedicated wire to the target lesion. Once activated, the crown orbits elliptically within the vessel lumen, employing sanding and pulsatile forces to effectively modify the calcified plaque [124]. This device has received commercial approval for the treatment of severely calcified *de-novo* lesions [125], but its use in the settings of ISR is currently off-label, and is only supported by limited observational data [126]. Compared to RA, OA offers advantages including the ability to treat larger diameter vessels using high-speed settings without increasing the burr size and provides bi-directional atheroablation (Table 4). Of note, both RA and OA are associated with a certain risk of burr entrapment and coronary perforation.

The excimer laser coronary angioplasty (ELCA) is a debulking technique that modifies and disrupts coronary plaques via heat and shock waves generated by ultraviolet spectrum wavelengths [127]. Evidence supporting ELCA’s superiority to BA in treating ISR is primarily derived from older observational studies [128, 129]. A small recent RCT demonstrated a lower rate of TLR at one year when ELCA was employed before DCB dilatation for treating BMS-ISR or DES-ISR [66] (Table 3). Thus, ELCA can be an attractive adjunctive therapy for heavy calcified, undilatable lesions. Despite these findings, there is no evidence supporting its routine use for ISR treatment. Additionally, this technique requires extra caution due to the risk of vessel perforation and no-reflow phenomenon (Table 4).

### 6.5 Repeat Stent Implantation

The initial superiority of BMS over BA for the treatment of *de-novo* coronary lesions [130] led to the hypothesis that BMS may also be a better option for managing ISR. However, the RIBS I (Restenosis Intra-stent: Balloon Angioplasty vs Elective Stenting) randomized trial failed to demonstrate better outcomes with BMS compared to BA [67]. The results of this trial, along with concerns about adding a new stent layer, led to the preferential use of BA over BMS implantation in the treatment of BMS-ISR. Currently, there is no randomized data available on the effectiveness of BMS for treating DES-ISR.

**Fig. 3.**
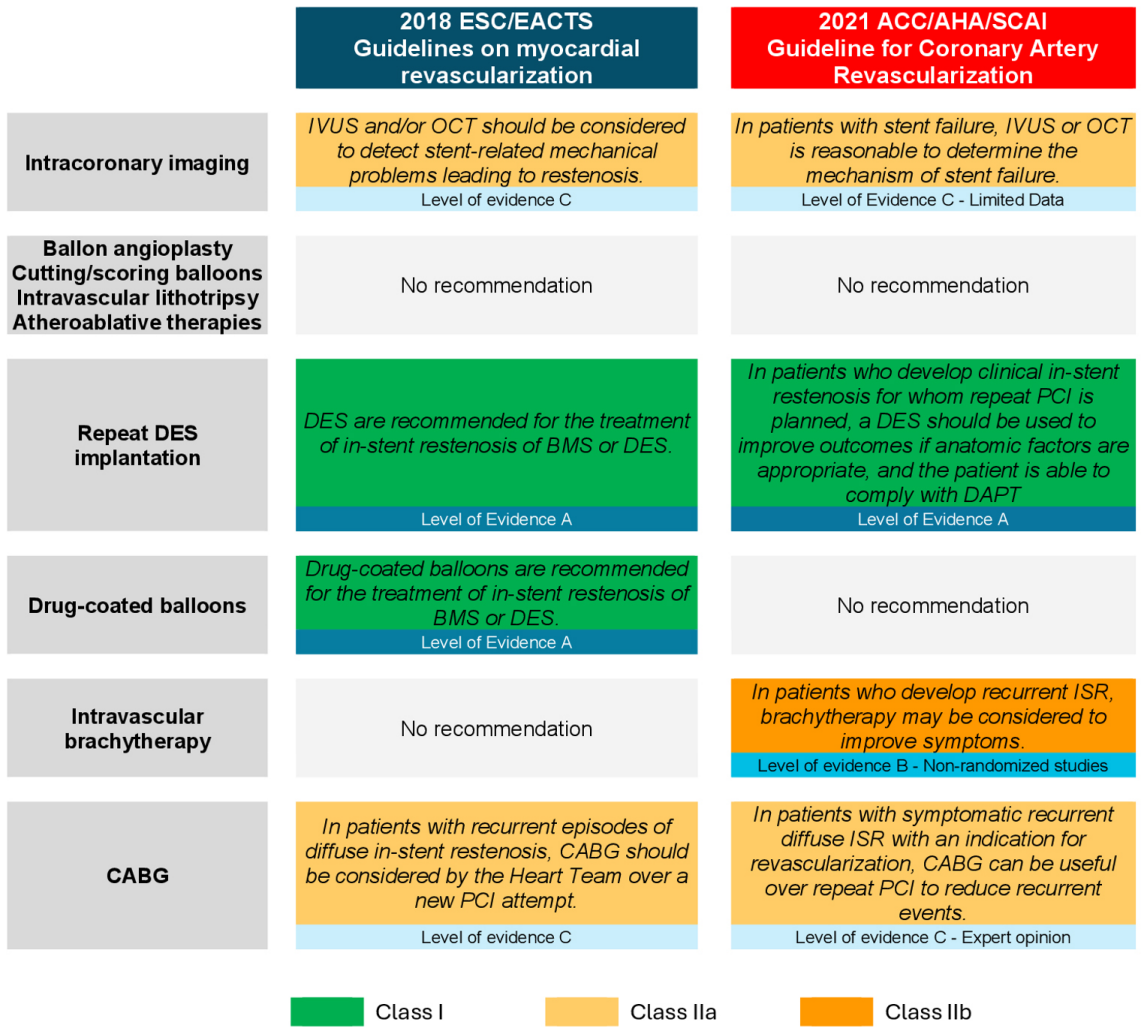
**Guidelines recommendations for the management of in-stent restenosis**. This figure summarizes the current guidelines from leading cardiovascular associations regarding ISR treatment. Key recommendations include the use of repeat DES implantation, supported by a Class I recommendation and Level of Evidence A. Abbreviations: ACC, American College of Cardiology; AHA, American Heart Association; BMS, bare metal stent; CABG, coronary artery bypass graft; DAPT, dual antiplatelet therapy; DES, drug eluting stent; EACTS, European association for Cardio-Thoracic surgery; ESC, European Society of Cardiology; ISR, in-stent Restenosis; IVUS, intravascular ultrasound; OCT, optical coherence tomography; PCI, percutaneous coronary intervention; SCAI, Society for Cardiovascular Angiography and Interventions.

The therapeutic approach to ISR treatment radically changed with the advent of DES. Several RCTs have shown that DES implantation yields superior angiographic and clinical outcomes compared to BA alone for treating both BMS- and DES-ISR (Table 3) [67, 68, 69]. In addition, DES were associated with better outcomes than intravascular brachytherapy (IVBT) among patients with BMS-ISR [70, 71, 131, 132]. The superiority of DES to other treatment modalities has been demonstrated in several meta-analyses [113, 114, 133]. Based on this evidence, the most recent guidelines for coronary revascularization recommend the use of repeat DES implantation for ISR management (Class of recommendation I, Level of Evidence A) (Fig. 3) [52, 53].

The hypothesis that antiproliferative drug resistance might play a role in the development of ISR was tested in the ISAR DESIRE-2 (Intracoronary Stenting and Angiographic Results: Optimizing Treatment of Drug-Eluting Stent In-Stent Restenosis) trial [72]. This RCT compared the outcomes of repeat implantation of hetero-DES (i.e., DES with a different antiproliferative drug than the one eluted by the restenotic stent) and homologous drug-eluting stents (homo-DES). This study demonstrated that both hetero-DES and homo-DES provided similar efficacy and safety in the treatment of ISR, raising questions about the role of antiproliferative drug resistance as a possible mechanism of ISR.

There are no direct randomized comparisons between newer generation and first-generation DES for ISR treatment. However, a prospective observational study and a subgroup analysis from the DAEDALUS study demonstrated improved outcomes with second-generation DES [49, 72, 133]. To date there is no conclusive evidence to suggest that any particular newer generation DES outperforms others in ISR treatment. This question was investigated only by a single RCT, which found no significant difference in major adverse cardiovascular events at three years between everolimus- and zotarolimus-based DES [73].

DES implantation is particularly useful in cases of stent fracture, geographic miss, stent edge dissections, or ISR extending beyond the stent edges (Table 4). Despite these advantages, DES usage has some drawbacks, such as the need for longer dual antiplatelet therapy (DAPT) and the potential complications arising from adding another stent layer. Particularly concerning is the placement of a third stent layer in cases of recurrent ISR, which has been associated with poorer outcomes [134, 135]. Therefore, placement of a third stent layer should generally be avoided.

### 6.6 Drug-Coated Balloons

DCBs consist of a semicompliant angioplasty balloon coated with a matrix containing an antiproliferative drug (i.e., paclitaxel, and more recently, sirolimus or biolimus) combined with an excipient or carrier. The excipient prevents the release of the antiproliferative drug into the bloodstream as the balloon is advanced to the lesion site. Once the balloon is inflated, it facilitates the drug transfer to the endothelial cells [136]. DCBs offer a distinct therapeutic advantage in the treatment of ISR by delivering an antiproliferative agent directly to the site of coronary lesions without the need to implant a new stent layer.

Several RCTs have demonstrated the superiority of paclitaxel coated ballon (PCB) compared to BA for the treatment of BMS- and DES-ISR [74, 75, 76, 77, 78] (Table 3). To date, the most extensive evidence concerns the SeQuent Please DCB (B. Braun Medical AG, Sempach, Switzerland), which is frequently used as a benchmark to test new DCBs [140]. A recent pivotal RCT conducted in the United States demonstrated a significant reduction in target lesion failure with PCB (Agent™, Boston Scientific, Marlborough, MA, USA) when compared to BA in 600 patients with ISR [79]. This study’s findings led to the approval of this device from the United States Food and Drug Administration for the treatment of ISR on March 1, 2024. Other types of DCBs with similar efficacy and safety are available outside the United States (Table 3). While most DCBs are coated with paclitaxel due to its high lipophilicity and optimal cellular uptake, recent RCTs have shown that newer generation sirolimus- and biolimus-coated balloons could become promising options for the treatment of ISR [80, 81, 82, 83].

RCTs comparing the efficacy of DCB with DES for ISR treatment yielded varied outcomes (Table 3). In one network meta-analysis, DCB was ranked as the second most effective device after DES with respect to angiographic outcomes and TLR [114]. Conversely, another network meta-analysis found that DCB and DES were both superior to other treatment modalities, but did not significantly differ from each other [113]. In the DAEDALUS study, an individual patient data meta-analysis of 10 RCTs with 1976 patients experiencing ISR, DES was found to be moderately more effective than DCB in reducing TLR rates after 3 years, however there were no differences in the composite outcomes of all-cause death, MI, or target lesion thrombosis [133]. Further analysis from the same dataset revealed that while DCB and DES showed similar efficacy and safety for BMS-ISR treatment, DCB was significantly less effective than repeat DES implantation for treating DES-ISR [137].

In summary, both DCB and DES are considered definitive strategies for ISR treatment, and their use is supported by European guidelines (Class I level of evidence A recommendation, Fig. 3) [53]. The choice between DCB and DES for treating ISR may vary depending on the specific type of ISR and the patient’s characteristics. These preferences are detailed further in the section titled “Practical Approach to ISR” (Table 4).

### 6.7 Intravascular Brachytherapy

IVBT refers to the intracoronary delivery of beta radiation using radioactive strontium-90 or yttrium 90, which inhibits fibroblast proliferation and subsequently neointimal growth at the coronary lesion site [84]. About 20 years ago, IVBT was approved for clinical use as adjunctive therapy following successful PCI with BA or BMS based on the improved angiographic and clinical outcomes observed in one large trial [84]. The findings of this trial were confirmed in five subsequent RCTs (Table 3) [85, 86, 87, 88, 89]. With the development of DES, the use of IVBT almost disappeared, as randomized studies demonstrated its inferiority in angiographic and clinical outcomes compared to DES for ISR treatment [70, 71, 131, 132]. Recently, IVBT re-emerged as a potential treatment option for multilayer ISR, although its application is only supported by observational studies [138, 139]. Consequently, the most recent European guidelines for coronary artery revascularization provide a Class IIb recommendation for IVBT in recurrent ISR cases, while the American guidelines do not provide any specific recommendation regarding IVBT use [52, 53] (Fig. 3). Of note, IVBT is available in only a few centers due to its complexity, issues related with radioprotection, and limited indications.

### 6.8 Coronary Artery Bypass Grafting

Coronary artery bypass grafting (CABG) involves surgically implanting a healthy artery or vein distally the diseased coronary segments to restore myocardial blood supply. CABG has been shown to be superior to PCI in reducing the need for repeat revascularization in several RCTs involving patients with left main or multivessel *de-novo* coronary lesions [52, 53]. Although there is no specific randomized data, CABG is considered a more effective treatment option for recurrent ISR than other strategies, such as the placement of a third stent layer, which is associated with TLR rates exceeding 40% at 1 year [135].

While CABG is a robust treatment for recurrent ISR, it comes with certain limitations. The procedure carries the risk of complications in the immediate postoperative period, especially in patients at high surgical risk. Additionally, performing CABG in coronary segments that have previously been treated with stents is not feasible (Table 4).

Guidelines recommend considering CABG in cases of recurrent ISR (Class IIa recommendation, Level of evidence C, Fig. 3) [52, 53]. In addition, CABG could be considered in patients with ISR located in the left main, ostial left artery descending artery (LAD), or in patients with multivessel disease [12, 57].

### 6.9 Medical Therapy

Implementing optimal secondary cardiovascular prevention strategies is pivotal to decrease the occurrence of adverse events in patients with coronary artery disease, including those with ISR [52, 53, 141]. A more potent antiplatelet therapy following PCI has been associated with a reduction in recurrent MI and stent thrombosis [52, 53, 142]. Additionally, lipid lowering therapy has been shown to improve prognosis by influencing plaque remodeling [143, 144]. However, specific evidence regarding secondary cardiovascular prevention in patients with ISR is lacking. Indeed, patients with ISR were either excluded or underrepresented in trials on antiplatelet or lipid lowering therapies after PCI. Since atherosclerosis is the most common mechanism of both *de-novo* coronary lesions and ISR, recommendations for secondary cardiovascular prevention strategies for patients with *de-novo* coronary lesions can generally be extended to patients with ISR.

For patients with high bleeding risk (HBR) who underwent DES implantation, it is recommended to de-escalate DAPT no later than 3 months after PCI extending up to 6 months following ACS [142]. In contrast, patients without HBR should receive DAPT for at least 6-months, or 12-month following ACS [142]. Conversely, patients at high ischemic risk might benefit from more potent antithrombotic regimens for a prolonged period of time (i.e., >12 months) [142]. Among patients undergoing BA or DCB only, DAPT can be safely shortened to 1 month after PCI. Additionally, secondary prevention targets for patients with ISR should include maintaining low-density lipoprotein cholesterol below 1.4 mmol/L, systolic blood pressure under 130 mmHg, and hemoglobin A1c (HbA1c) levels below 53 mmol/mol (7%). These medical targets are complemented by advocating for smoking cessation and promoting improvements in lifestyle choices [52, 141].

### 6.10 Practical Approach to ISR

**Fig. 4.**
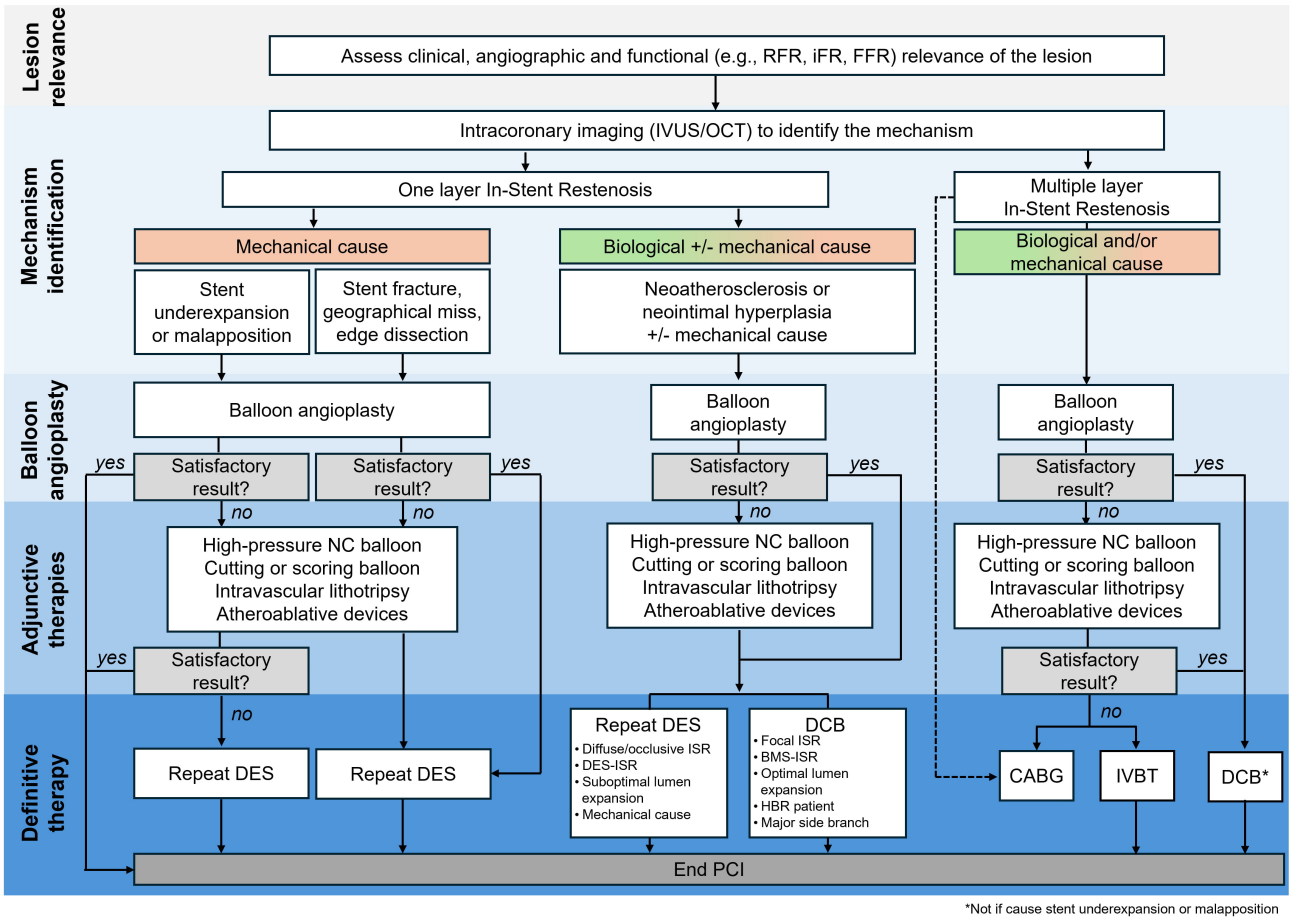
**Algorithm for the management of in-stent restenosis**. Abbreviations: BMS, bare metal stent; CABG, coronary artery bypass graft; DCB, drug-coated balloon; DES, drug eluting stent; iFR, instantaneous wave-free ratio; ISR, in-stent restenosis; IVUS, intravascular ultrasound; FFR, fractional flow reserve; HBR, high bleeding risk; IVBT, intravascular brachytherapy; NC, non-compliant; OCT, optical coherence tomography; PCI, percutaneous coronary intervention; RFR, resting full cycle ratio. *Not if the cause of ISR is stent underexpansion or malapposition.

The general treatment approach to ISR doesn’t significantly differ from that of de novo lesions, even though the presence of one or more stent layers poses additional challenges. First, the indication for revascularization of ISR should be carefully evaluated based not only on angiographic assessment, but also on clinical symptoms and hemodynamic relevance [145] (Fig. 4). It is important to note that routine revascularization of intermediate coronary stenosis, which is not associated with anginal symptoms or significant hemodynamic flow impairment, does not yield any clinical benefits [145].

The second step in ISR management comprises a morphological characterization of the restenotic lesion and the identification of any underlying mechanisms contributing to ISR. The type of restenotic stent (BMS or DES), the extent of the ISR, the presence of mechanical issues (e.g., stent underexpansion, stent fracture, geographic miss), and the identification of biological patterns (e.g., neointimal hyperplasia, neoatherosclerosis, calcifications) are essential factors to inform ISR treatment (Fig. 4). Failure to correct mechanical issues can lead to a higher risk of recurrent ISR [12, 25, 41, 57]. Use of intracoronary imaging – OCT or IVUS – is recommended to characterize the ISR [25, 52, 53] (Fig. 3), while radiological stent enhancement might be useful for identifying fractures or stent underexpansion [41, 45, 46].

Treatment of ISR typically involves a stepwise approach, starting with lesion preparation using BA. If the outcomes post-BA are unsatisfactory, adjunctive therapies (i.e., UHPNC balloon, CB or SB, IVL, and atheroablative devices) may be employed. Most ISR require a definitive treatment with DCB or DES (Fig. 4). While only DCB and DES have demonstrated efficacy for ISR treatment [113, 114], the sequential use of different modalities is likely the best approach to maximize acute gain and minimize late lumen loss [41, 57].

BA is the first step in ISR treatment regardless of the underlying cause or number of stent layers present in the lesion. The goal of BA is to achieve a residual stenosis of 30% or less, while avoiding significant coronary dissections (i.e., longitudinal extension >2 mm, lateral extension >60º or involvement of the media or adventitia). To minimize the risk of balloon slippage during the procedure, the use of BA techniques with non-slip elements, such as CB, or SB, is recommended.

While ISR due to stent underexpansion or malapposition can be treated with BA alone, UHPNC balloon or DES implantation should be considered if the acute lumen gain is unsatisfactory. Conversely, complete correction of mechanical causes such as stent fracture, geographic miss or edge dissection always requires DES implantation. A persistent diameter stenosis greater than 30% after BA in ISR attributed to mechanical causes, often indicates the presence of concomitant biological causes (e.g., calcification), which may require adjunctive therapies.

In cases of NIH or non-calcified neoatherosclerosis with unsatisfactory results following BA treatment, utilizing an UHPNC balloon may facilitate better lesion expansion. Additionally, CB or SB may improve the penetration of antiproliferative medications into the lesion, potentially improving therapeutic outcomes. For calcified lesions, IVL is particularly beneficial by specifically targeting and breaking down calcified deposits. Atheroablative devices may be considered as a second-line strategy to remove excess neointimal or neoatherosclerotic tissue.

After achieving optimal lesion preparation, the definitive treatment of ISR stemming from biological causes includes either repeat implantation of DES or the application of a DCB. DES is preferred for managing diffusive or occlusive ISR, DES-ISR, suboptimal lumen expansion, significant residual dissection after lesion preparation, and in the presence of concomitant mechanical issues (Fig. 4). However, the main disadvantage of using DES is the addition of a new stent layer within the lesion.

Conversely, DCB is more appropriate for focal ISR, BMS-ISR, and scenarios where the lesion preparation is satisfactory. It is also favored when the major side branch is at risk of reduced flow in case of repeat DES implantation, and for HBR patients who cannot tolerate longer DAPT regimens.

A different algorithm should be applied in the case of multilayer ISR (i.e., ≥2 stent layers). In this situation a third stent layer should be avoided due to the high risk of ISR recurrence [134, 135]. The preferred definitive therapies in such cases include DCB which can deliver antiproliferative drugs directly to the lesion without introducing additional stent layers. If the results post-lesion preparation remains unsatisfactory, other options like CABG or IVBT should be considered [41, 139].

Additional considerations may be needed in specific ISR cases. Treatment of in-stent CTO or ISR occurring in SVG, or in the left main coronary artery often results in poorer outcomes compared to *de-novo* lesions, mostly due to higher rates of TLR [146, 147, 148, 149]. There is a notable scarcity of evidence regarding the optimal treatment approaches for ISR in these specific settings.

Observational studies suggest that the technical and procedural success rates for PCI in CTO-ISR are comparable to those for *de-novo* CTO [149, 150, 151]. Conversely, when dealing with ISR in a SVG, the preferred strategy is to treat the native vessel rather than the restenotic SVG, owing to the high incidence of adverse events associated with graft treatment [152].

When addressing ISR of the left main coronary artery, treatment options include PCI using either DES or DEB. Observational studies suggest that the immediate outcomes of DES and DEB in this context are comparable [153, 154]. Despite these findings, the long-term outcomes of PCI in treating ISR located in the left main coronary artery are often less satisfactory, prompting consideration of CABG in eligible candidates [155].

## 7. Future Directions

Four ongoing RCTs will provide additional insights for ISR treatment (Table 5). The OPEN ISR (NCT04862052) trial is examining the effectiveness of the PCB Emperor (AR Baltic Medical, Vilnius, Lituania) and sirolimus coated-balloon (SCB) Magic Touch (Concept Medical, Tampa, FL, USA), compared with the Xience everolimus-eluting stent (EES; Abbott, IL, USA) in patients with DES-ISR. Another trial is comparing the outcomes of treating DES-ISR with the SCB Magic Touch (Concept Medical, Tampa, FL, USA) versus BA alone (NCT05908331). The ISAR DESIRE 5 trial (NCT05544864) is comparing the performance of any DCB with Xience (Abbott, Illinois, USA) EES in patients with DES-ISR. Finally, The SCB SELUTION SLR™ (MedAlliance, Nyon, Switzerland) is being evaluated against the standard of care (Zotarolimus-eluting stent or EES or BA) in patients with DES- or BMS-ISR (NCT04280029).

**Table 5.** **Ongoing randomized clinical trials on devices for the treatment of ISR**.

Trial name	Sample size	Number clinical trial	Population	Experimental group	Control group	Primary endpoint	Planned study completion
OPEN ISR	150	NCT04862052	DES-ISR	Emperor PCB	EES (Xience)	Target vessel MI	Jan 2025
				Magin touch SCB		Target vessel failure, TLR at 6-months	
No name*	492	NCT05908331	DES-ISR	Magin touch SCB	BA	Target lesion failure at 12-months	Sep 2025
ISAR DESIRE 5	376	NCT05544864	DES-ISR	Any DCB	EES (Xience)	Composite of all-cause death, MI or TLR at 24 months	Sep 2026
SELUTION SLR™ 014 ISR	418	NCT04280029	DES-ISR or BMS- ISR	SELUTION SLR™ SCB	ZES or EES or BA	Target lesion failure at 12-months	Nov 2027

BA, balloon angioplasty; BMS, bare metal stents; DCB, drug coated balloon; DES, drug eluting stent; EES, everolimus eluting stent; ISR, in-stent restenosis; MI, myocardial infarction; PCB, paclitaxel coated balloon; SCB, sirolimus coated balloon; TLR, target lesion revascularization; ZES, zotarolimus-eluting stents. *Title: MagicTouch Sirolimus-coated Balloon for Treatment of In-Stent Restenosis in Coronary Artery Lesions. Related clinical trial information query: https://clinicaltrials.gov/.

## 8. Conclusions

ISR remains a significant challenge in interventional cardiology, occurring at an annual rate of 1–2% following DES implantation. The application of intravascular imaging techniques is crucial for identifying the underlying mechanisms of ISR and informing the management strategy. While stent underexpansion or malapposition can be effectively addressed with BA alone, other mechanical causes of ISR, such as NIH, and neoatherosclerosis, generally require treatment with repeat DES implantation or the use of DCB. The decision between these two treatment modalities, as well as the application of adjunctive therapies, should be tailored based on lesion and patient’s characteristics. For cases of recurrent ISR, options such as DCB, surgical revascularization, or IVBT are recommended. Despite these advancements, there remains a critical need for additional randomized controlled trials evaluating the effectiveness of specific devices, whether used alone or in combination, for the treatment of ISR, and for studies assessing the effects of targeted secondary prevention strategies for patients experiencing ISR.

## Abbreviations

ACC/AHA/SCAI, American College of Cardiology**/**American Heart Association/Society for Cardiovascular Angiography and Interventions; ACS, acute coronary syndrome; BMS, bare metal stent; CABG, coronary artery bypass grafting; CB, cutting balloon; CTO, chronic total occlusion; DAPT, dual antiplatelet therapy; DCB, drug-coated balloon; DES, drug eluting stent; ELCA, Excimer Laser Coronary Angioplasty; ESC/EACTS, European Society of Cardiology/European Association for Cardio-Thoracic Surgery; ISR, in-stent restenosis; IVBT, intravascular brachytherapy; IVL, intravascular lithotripsy; NIH, neointimal hyperplasia; OA, orbital atherectomy; OCT, optical coherence tomography; PCI, percutaneous coronary intervention; RA, rotational atherectomy; RCT, randomized clinical trial; SB, scoring balloon; SCB, sirolimus coated balloon; SVG, saphenous vein graft; TLR, target lesion revascularization; UHPNC, ultra-high-pressure noncompliant.
